# ZmNPSN13 Potentiates Maize Rough Dwarf Disease Upon Recruitment by the RBSDV P7‐1 Effector

**DOI:** 10.1111/mpp.70277

**Published:** 2026-05-19

**Authors:** Suining Deng, Yixiao Qin, Baoshen Liu, Siqi Jiang, Tao Zhong, Qingcai Liu, Jianju Liu, Yuanliang Liu, Manman Li, Yanmei Li, Mingzhu Yan, Qian Li, Mingliang Xu

**Affiliations:** ^1^ State Key Laboratory of Maize Bio‐Breeding/College of Agronomy and Biotechnology/National Maize Improvement Center China Agricultural University Beijing China; ^2^ College of Agronomy/State Key Laboratory of Crop Biology Shandong Agricultural University Taian China

**Keywords:** maize rough dwarf disease, phytohormone, plant SNARE protein, ZmGDIα‐hel

## Abstract

Maize rough dwarf disease (MRDD), caused by fijiviruses, poses a major threat to global maize production, with rice black‐streaked dwarf virus (RBSDV) being the predominant pathogen in Asia. The viral effector P7‐1 initiates MRDD by hijacking the host susceptibility factor ZmGDIα, while its reduced affinity for the *helitron*‐inserted variant ZmGDIα‐hel underlies recessive resistance. Here, we identified the Qb‐SNARE protein ZmNPSN13 as a novel host target of P7‐1. ZmNPSN13 functions as a central hub, interacting with both ZmGDIα and the gibberellin‐inactivating enzyme ZmGA2ox7.3. During infection, P7‐1 recruits these host components into a pathogenic complex that disrupts phytohormone homeostasis, thereby driving MRDD development. Notably, knockout of *ZmNPSN13* restored hormonal balance and enhanced viral resistance comparable to that achieved by replacing *ZmGDIα* with *ZmGDIα‐hel*. Our work uncovers a mechanism by which a virus exploits SNARE‐mediated vesicle trafficking to subvert host immunity, establishing *ZmNPSN13* as a promising target for engineering MRDD‐resistant maize cultivars.

## Graphical abstract

ZmNPSN13 serves as an additional host target of P7‐1 and forms a pathogenic complex with ZmGDIα and ZmGA2ox7.3, which is disrupted by replacing ZmGDIα with ZmGDIα‐hel. Knockout of *ZmNPSN13* restores hormonal balance and enhances resistance to MRDD.

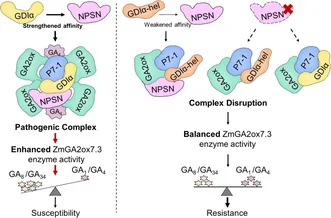

## 1 Introduction

Maize rough dwarf disease (MRDD) is a devastating viral disease that leads to severe yield losses, ranging from 30% to 100% (Yang et al. 2017). The disease is caused by several species of the genus *Fijivirus* and primarily transmitted by the small brown planthopper ( *Laodelphax striatellus* ), with rice black‐streaked dwarf virus (RBSDV) being the most predominant species in Asia (Liu et al. 2020; Xu et al. 2023). Current control measures, such as adjusting sowing schedules and applying agrochemicals, are resource‐intensive, environmentally unsustainable and often ineffective (Wu et al. 2020). Therefore, identifying MRDD‐resistance genes, elucidating their underlying mechanisms and developing resistant cultivars provide a more sustainable and effective solution to disease management.

Vesicle trafficking, orchestrated by a sophisticated molecular machinery comprising ADP ribosylation factors (ARFs), small GTPases, tethering factors and soluble N‐ethylmaleimide‐sensitive factor attachment protein receptor (SNARE), plays a central role in regulating protein abundance, localization and inter‐organelle transport, thereby ensuring proper cellular function (Gu et al. 2017). A key regulator in this process is the GDP dissociation inhibitor (GDI), which extracts prenylated Rab proteins from membranes and shuttles them as soluble cytosolic complexes, thereby facilitating Rab protein recycling (Gavriljuk et al. 2013; Kim et al. 1999; Seabra et al. 2002).

During viral infection, plant vesicles are frequently hijacked to form viral replication factories or to aid in viral particle transport (Laliberté and Zheng 2014; Movahed, Sun, et al. 2019). For instance, the *Turnip mosaic virus* (TuMV) 6K2 protein remodels host membranes to generate vesicles that encapsulate viral replication complexes (VRCs) (Cotton et al. 2009; Grangeon et al. 2012). These vesicles are then transported along actin filaments to plasmodesmata (PD) with the aid of the motor protein RHD3, facilitating cell‐to‐cell viral spread (Grangeon et al. 2012, 2013; Jiang et al. 2015; Movahed, Sun, et al. 2019). Moreover, extracellular vesicles released from *Nicotiana benthamiana* during TuMV infection contain both viral RNA and proteins (Movahed, Cabanillas, et al. 2019). Similarly, the *Tobacco mosaic virus* replication protein interacts with NtGDI2 to subvert vesicle trafficking to promote viral infection (Kramer et al. 2011).

SNARE complexes play a fundamental role in mediating vesicle fusion with target membranes (Zhu et al. 2019). Based on subcellular localization, SNARE proteins are classified as t‐SNAREs, associated with target membranes, or v‐SNAREs, associated with transport vesicles (Kwon et al. 2020). They are further categorized by conserved residues: Q‐SNAREs contain glutamine, while R‐SNAREs contain arginine (Gu et al. 2017; Kwon et al. 2020; Yun and Kwon 2017). Q‐SNAREs are subdivided into Qa‐, Qb‐, Qc‐ and Qbc‐SNAREs (Bock et al. 2001; Gu et al. 2017; Kwon et al. 2020; Won and Kim 2020). The composition of SNARE complexes depends on the fusion type: intracellular membrane fusion requires a tetrameric complex of Qa‐, Qb‐, Qc‐ and R‐SNAREs, while exocytosis involves a ternary complex of Qa‐, Qbc‐ and R‐SNAREs (Gu et al. 2017; Jahn and Scheller 2006). In plants, the VAMP729 (R‐SNARE)‐SYP22 (Qa‐SNARE) complex likely regulates defence responses by modulating BRI1 abundance at the plasma membrane (Zhu et al. 2019).

Pathogens frequently exploit SNARE‐mediated vesicle trafficking for pathogenesis. For example, Southern rice black‐streaked dwarf virus hijacks vesicles in insect gut epithelial cells by interacting with VAMP7 (R‐SNARE) and Vti1a (Qb‐SNARE) to facilitate viral transport (Zhang et al. 2021). Conversely, SNARE proteins contribute to host resistance. For instance, TaNPSN11 (Qb‐SNARE) localizes to fungal invasion sites to inhibit hyphal elongation and enhance wheat resistance to stripe rust (Wang et al. 2014). Similarly, ShNPSN11 triggers vesicle trafficking‐dependent defence responses against powdery mildew in tomato (Lian et al. 2020). By contrast, TuMV subverts SNARE function by recruiting the Qb‐SNARE Sec22 through its 6K2 protein, thereby disrupting endoplasmic reticulum (ER)‐Golgi vesicle fusion and promoting viral spread (Cabanillas et al. 2018).

In our previous study, we identified a naturally occurring resistance gene, *ZmGDIα‐hel*, arising from a *helitron* insertion into *ZmGDIα*, that confers recessive quantitative resistance to MRDD (Liu et al. 2020). We further demonstrated that the viral effector P7‐1 targets both the susceptibility factor ZmGDIα and the host enzyme ZmGA2ox7.3, forming hetero‐oligomers that disrupt gibberellin (GA) and auxin/cytokinin (IAA/CK) homeostasis, thereby promoting MRDD development (Deng et al. 2024). Importantly, the ZmGDIα‐hel variant exhibits a weakened interaction with P7‐1, alleviating RBSDV‐induced hormonal imbalance and conferring MRDD resistance (Deng et al. 2024). Despite these insights, the precise molecular mechanisms underlying this resistance remain unclear.

In this study, we identify ZmNPSN13, a Qb‐SNARE protein, as a critical host factor for RBSDV infection through its interactions with both the viral effector (P7‐1) and the host proteins ZmGA2ox7.3 and ZmGDIα/ZmGDIα‐hel. This discovery deepens our understanding of the molecular mechanisms underlying MRDD resistance and provides a foundation for breeding resistant maize varieties.

## 2 Results

### 2.1 ZmNPSN13 Binds More Tightly to ZmGDIα and P7‐1 Than to ZmGDIα‐hel

**FIGURE 1:**
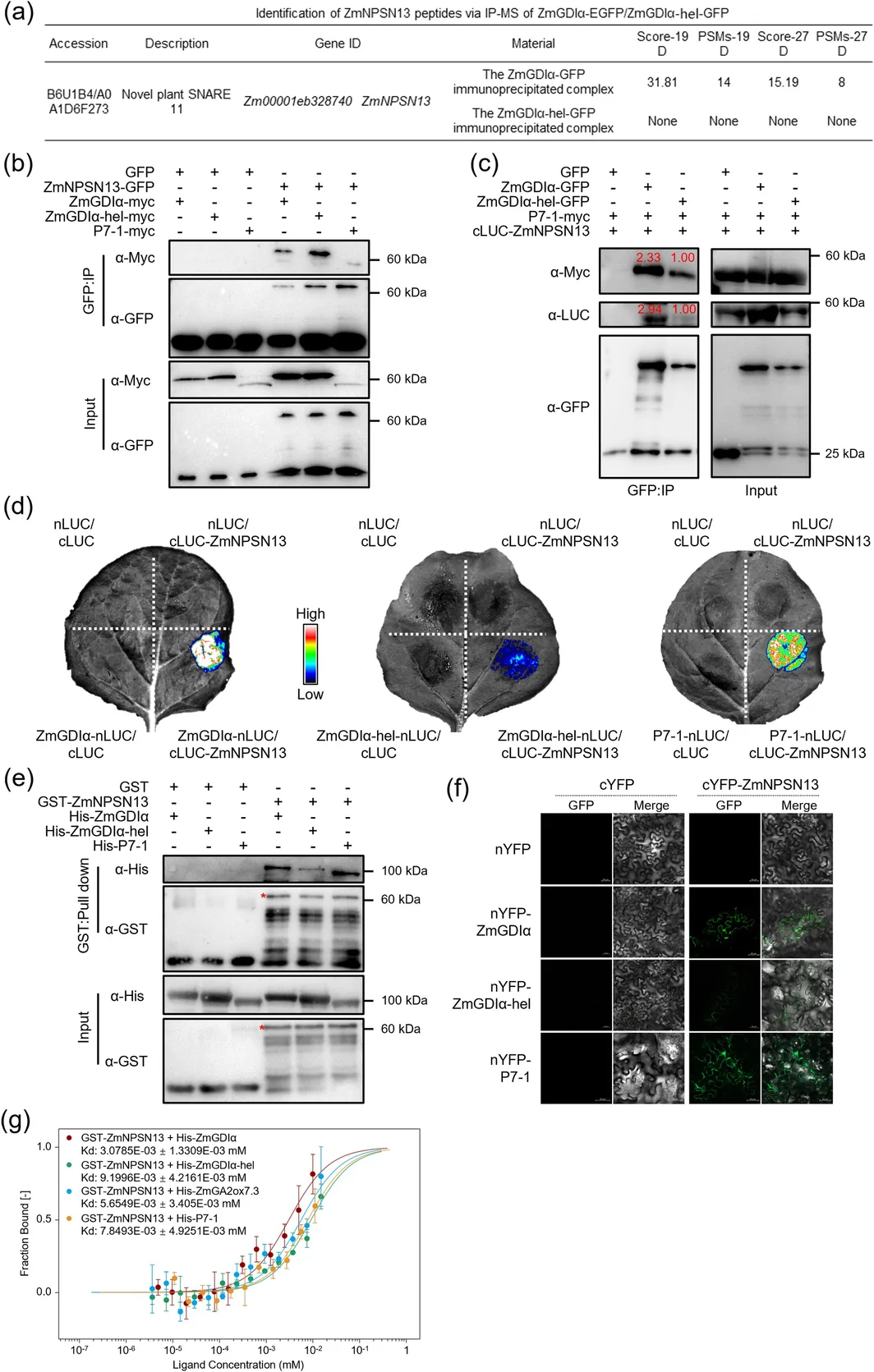
Identification of ZmNPSN13 and characterization of its interactions with host proteins ZmGDIα/ZmGDIα‐hel and viral effector P7‐1. (a) Identification of ZmNPSN13 peptides in ZmGDIα‐EGFP and ZmGDIα‐hel‐EGFP immunoprecipitated complex by immunoprecipitation‐mass spectrometry (IP‐MS). (b) Confirmation of the interactions of ZmNPSN13 with ZmGDIα, ZmGDIα‐hel and P7‐1 using a co‐immunoprecipitation (Co‐IP) assay. (c) Interaction among ZmNPSN13, P7‐1 and ZmGDIα (or ZmGDIα‐hel) in Co‐IP assay. (d) Validation of the interactions of ZmNPSN13 with ZmGDIα, ZmGDIα‐hel or P7‐1 using an in vivo split‐luciferase complementation (SLC) assay. The colour scale depicts the intensity of the luminescence signals. (e) Confirmation of the interactions of ZmNPSN13 with ZmGDIα, ZmGDIα‐hel and P7‐1 using an in vitro glutathione S‐transferase (GST) pull‐down assay. (f) Confirmation of the interactions of ZmNPSN13 with ZmGDIα, ZmGDIα‐hel and P7‐1 using an in vivo bimolecular fluorescence complementation (BiFC) assay. Reconstituted YFP fluorescence signals were visualized using confocal microscopy and captured at 72 h post‐inoculation (hpi). Scale bar, 50 μm. (g) Microscale thermophoresis (MST) assays were performed to measure the binding affinities of ZmGDIα, ZmGDIα‐hel, ZmGA2ox7.3 and P7‐1 to ZmNPSN13. Each binding assay was repeated independently at least three times, and bars represent standard deviations. *K*_d_ is equal to the dissociation constant (*n* ≥ 3).

We generated transgenic maize lines overexpressing either the susceptible (*ZmGDIα‐GFP*) or resistant (*ZmGDIα‐hel‐GFP*) fusion constructs. Immunoprecipitation‐mass spectrometry (IP‐MS) identified ZmNPSN13, a previously uncharacterized plant SNARE protein, as a specific interactor of ZmGDIα‐GFP but not of ZmGDIα‐hel‐GFP (Figure 1a). To determine whether ZmNPSN13 contributes to the assembly of a pathogenic complex, we examined its interactions with ZmGDIα, ZmGDIα‐hel and the viral effector P7‐1. Co‐immunoprecipitation (Co‐IP) assays showed that ZmNPSN13 interacted with all three proteins (Figure 1b). In *N. benthamiana* leaves, both P7‐1 and ZmNPSN13 were co‐immunoprecipitated by ZmGDIα and ZmGDIα‐hel, with consistently stronger interactions observed with ZmGDIα (Figure 1c). Split‐luciferase complementation (SLC) assays further demonstrated higher luminescence when cLUC‐ZmNPSN13 was paired with ZmGDIα‐nLUC or P7‐1‐nLUC than with ZmGDIα‐hel‐nLUC (Figure 1d). These results were corroborated by in vitro pull‐down and bimolecular fluorescence complementation (BiFC) assays, which confirmed preferential binding of ZmNPSN13 to ZmGDIα and P7‐1 over ZmGDIα‐hel (Figure 1e,f). Microscale thermophoresis (MST) analysis showed that ZmNPSN13 bound to both ZmGDIα and P7‐1 with high affinity. As expected, its binding to ZmGDIα‐hel was comparatively weaker (Figure 1g). Quantitative SLC assays further revealed the strongest luciferase activity for the ZmNPSN13‐ZmGDIα interaction, followed by the ZmNPSN13‐P7‐1, while activity with ZmGDIα‐hel was markedly weaker (Figure S1a–g).

To determine whether ZmNPSN13 is associated with vesicle trafficking, we co‐transformed ZmNPSN13‐GFP with AtMAN1‐mCherry, a marker for the ER, Golgi apparatus and plasma membrane, in *Nicotiana benthamiana* leaves. Subcellular localization analysis revealed clear colocalization of ZmNPSN13‐GFP with AtMAN1‐mCherry (Figure S2a). Treatment with brefeldin A (BFA), a vesicle trafficking inhibitor that disrupts the Golgi apparatus and retrograde transport, induces the formation of BFA compartments (BFA body). In BFA‐treated cells. ZmNPSN13‐GFP accumulated in these BFA bodies (Figure S2b). Notably, BFA treatment also induced colocalization of P7‐1‐mCherry with ZmNPSN13‐GFP within BFA bodies (Figure S2c). Together, these results indicated that ZmNPSN13 is involved in ER‐to‐Golgi vesicle membrane transport and that P7‐1 hijacks host vesicle trafficking via ZmNPSN13 to evade immunity.

Together, these results demonstrate that ZmNPSN13 forms a more stable complex with P7‐1/ZmGDIα than with P7‐1/ZmGDIα‐hel. Because NPSN family members have been implicated in plant immunity (Inada and Ueda 2014; Wang et al. 2014), ZmNPSN13 probably plays a critical role in mediating MRDD resistance.

### 2.2 ZmNPSN13 Negatively Regulates MRDD Resistance

**FIGURE 2:**
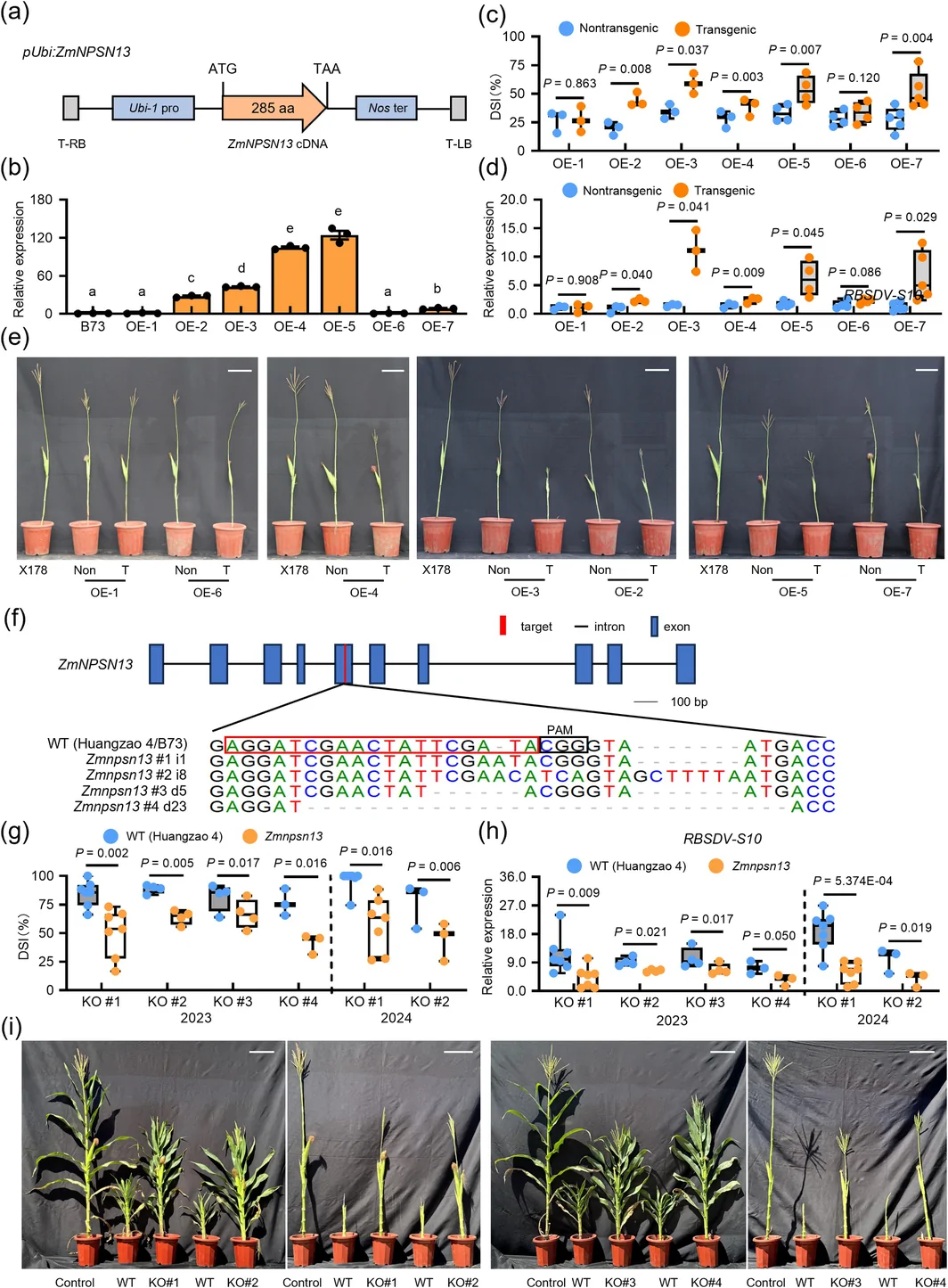
*ZmNPSN13* promotes maize rough dwarf disease (MRDD) susceptibility. (a) Schematic diagram of the *ZmNPSN13* overexpression construct *pUbi:ZmNPSN13*. (b) Relative expression levels of *ZmNPSN13* in transgenic lines. The B73 recipient line served as a negative control. (c) Disease severity index (DSI) values of T_2_F_1_BC_2_ progeny at 60 days post‐inoculation (dpi). DSI values were estimated for both transgenic and non‐transgenic T_2_F_1_BC_2_ plants. Each dot represents the DSI value of a biological replicate. (d) Relative expression levels of RBSDV‐*S10* in T_2_F_1_BC_2_ plants at 60 dpi. Leaf tissues were sampled three times independently as three biological replicates. (e) Resistance performance of T_2_F_1_BC_2_ progeny at 60 dpi. Scale bar, 20 cm. (f) Schematic representation of the *ZmNPSN13* genomic structure and the sgRNA target site (located in the fifth exon) used for CRISPR/Cas9 editing. The wild‐type sequence (Huangzao 4/B73) is displayed at the top. The target sites and PAM sequences are highlighted in red and grey, respectively. Edited sequences of four homozygous knockout lines (*Zmnpsn13* #1–4) are shown below. (g) DSI values of T_2_F_2_BC_2_ plants (wild‐type vs. homozygous *Zmnpsn13* lines) at 60 dpi. Each dot represents the DSI value of a biological replicate. (h) Relative RBSDV *S10* expression in T_2_F_2_BC_2_ plants at 60 dpi (three independent replicates; *Zmnpsn13* #1 transgenic plants as control). (i) Resistance performance of T_2_F_2_BC_2_ progeny at 60 dpi. Scale bar, 25 cm. In (b), data represent mean ± SEM from three replicates (one‐way ANOVA with Tukey's test). In (c, d, g, h), box plots are displayed. Error bars represent the maximum and minimum values, central lines indicate medians and box edges denote the 25th and 75th percentiles. Statistical significance was determined by a two‐tailed Student's t test (c, d, g, h).

To investigate the role of ZmNPSN13 in MRDD resistance, we introduced the overexpression construct *pUbi:ZmNPSN13* into the susceptible recipient line B73 (Figure 2a). Among seven independent T_2_ transgenic lines, significant upregulation of *ZmNPSN13* was observed in all lines except OE‐1 and OE‐6 (Figure 2b, Table S2). The wild‐type *ZmGDIα* functions as a dominant susceptibility allele for RBSDV infection, whereas the *ZmGDIα‐hel* variant represents a recessive resistance allele. The transgenic recipient line B73 carries the endogenous *ZmGDIα* and is highly susceptible to MRDD (Deng et al. 2024; Liu et al. 2020). Consequently, these transgenic lines were crossed with the resistant line X178 and subsequently backcrossed twice to generate T_2_F_1_BC_2_ progeny. Individuals carrying homozygous resistant *ZmGDIα‐hel* alleles were selected and then inoculated with RBSDV. Transgenic plants overexpressing *ZmNPSN13* exhibited significantly higher disease severity index (DSI) and elevated RBSDV *S10* transcript levels compared to non‐transgenic controls, except for OE‐1 and OE‐6 (Figure 2c–e).

To further assess its function, we generated four *ZmNPSN13* knockout mutants (*Zmnpsn13* #1–4) in the B73 background using CRISPR/Cas9 gene‐editing technology. These mutants harboured distinct genetic modifications in the fifth exon of *ZmNPSN13*, including insertions (1‐bp and 8‐bp) and deletions (5‐bp and 23‐bp) (Figure 2f). The mutants were crossed with the susceptible line Huangzao 4, then backcrossed twice and selfed to produce T_2_F_2_BC_2_ progeny. From this progeny, individuals that were homozygous for the susceptible *ZmGDIα* alleles were selected and inoculated with RBSDV. All four knockout lines displayed significantly reduced DSI values and lower RBSDV *S10* expression compared to wild‐type (WT) controls (Figure 2g–i). These results collectively demonstrate that ZmNPSN13 facilitates RBSDV infection, thereby contributing to MRDD development.

### 2.3 RBSDV‐Induced Transcriptome Reprogramming in *Zmnpsn13* #1 Plants

**FIGURE 3:**
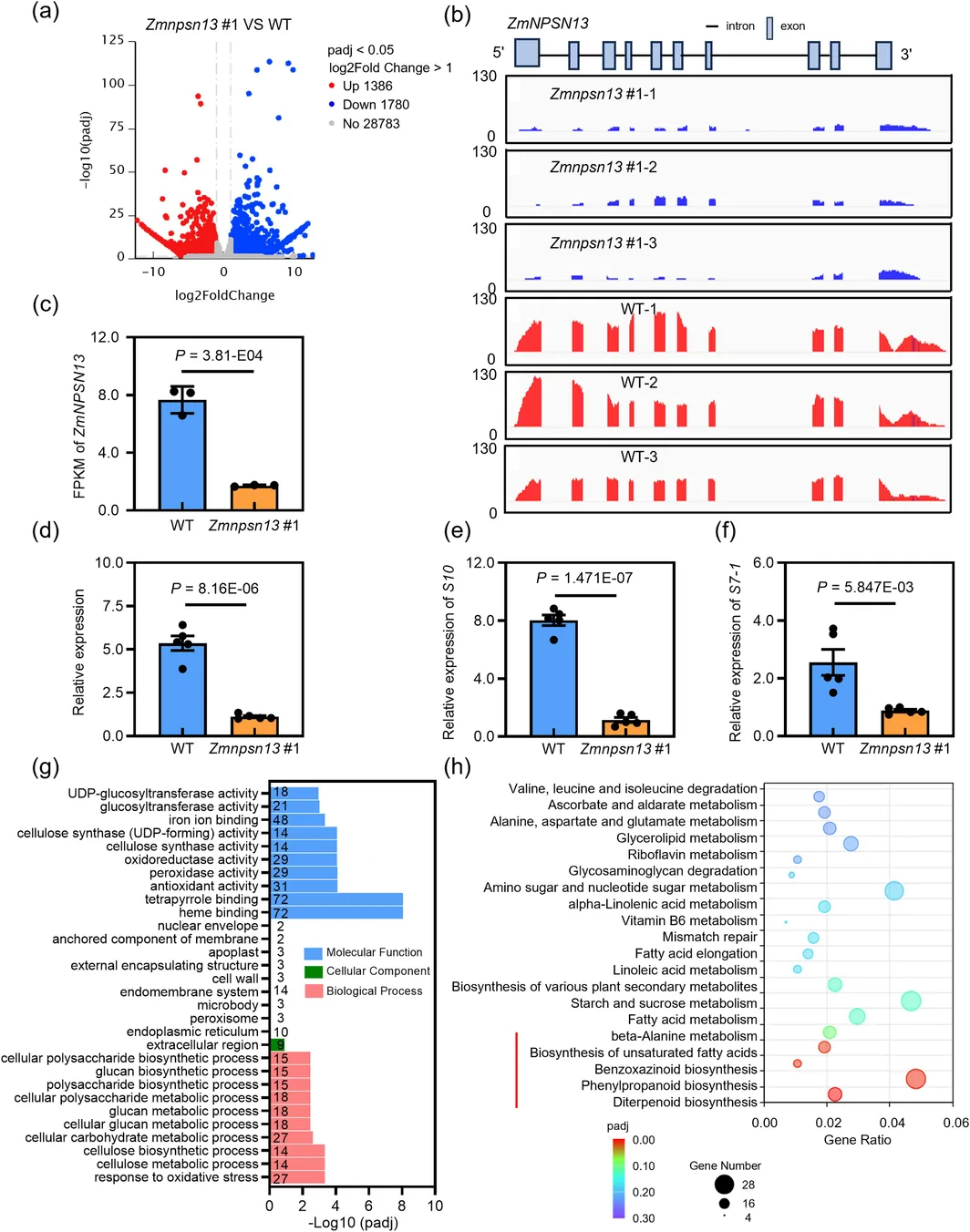
Transcriptome reprogramming in the *ZmNPSN13* knockout mutant in response to rice black‐streaked dwarf virus (RBSDV) infection. (a) A volcano plot depicting differentially expressed genes (DEGs) in *Zmnpsn13* #1 versus wild‐type (WT) plants at 24 days post‐inoculation (dpi). Genes with significant downregulated are represented by blue dots, those with significant upregulated by red dots and those without significant changes by grey dots. (b) Transcript abundance of *ZmNPSN13* in *Zmnpsn13* #1 compared to WT plants. The gene structure of *ZmNPSN13* is provided at the top. (c) FPKM (fragments per kilobase million) values of *ZmNPSN13* transcripts in *Zmnpsn13* #1 versus WT. (d) Relative expression levels of *ZmNPSN13* as verified by reverse transcription‐quantitative PCR. (e, f) Relative expression levels of RBSDV *S10* and *S7‐1* in *Zmnpsn13* #1 versus WT plants at 24 dpi. A biological replicate in WT served as a control for normalization. (g) GO classification of DEG‐enriched processes, with molecular function, cellular process and biological process represented by blue, green and red bars, respectively. (h) KEGG analysis of DEG‐enriched pathways in *Zmnpsn13* #1 following RBSDV inoculation. Circle sizes represent the number of DEGs. The red vertical line indicates the five most significantly enriched pathways (*p* < 0.05). In (c), data are presented as means ± SD. In (d, e, f), data are presented as means ± SEM. Statistical significance was determined by a two‐tailed Student's t test.

To elucidate the molecular mechanisms underlying *ZmNPSN13*‐mediated regulation of RBSDV infection, we performed transcriptome sequencing (RNA‐seq) on *Zmnpsn13* #1 mutant and WT plants at 24 days post‐inoculation (dpi). Comparative transcriptomic analysis identified 3166 differentially expressed genes (DEGs) between *Zmnpsn13* #1 and WT plants (*p* < 0.05, log_2_ fold change > |1|), including 1386 upregulated and 1780 downregulated genes (Figure 3a). As expected, both RNA‐seq and reverse transcription‐quantitative real‐time PCR (RT‐qPCR) analyses confirmed markedly reduced *ZmNPSN13* expression in *Zmnpsn13* #1 compared to WT plants (Figure 3b–d). Notably, while viral *S7‐1* and *S10* segments accumulated dramatically in WT plants, they were barely detectable in the *Zmnpsn13* #1 mutant (Figure 3e,f).

Gene Ontology (GO) analysis revealed that *ZmNPSN13*‐regulated DEGs are predominantly enriched in molecular functions (e.g., heme and tetrapyrrole binding) and biological processes (including response to oxidative stress and cellular carbohydrate metabolic processes) (Figure 3g). Kyoto Encyclopedia of Genes and Genomes (KEGG) pathway analysis highlighted significant enrichment in diterpenoid, phenylpropanoid, benzoxazinoid, and unsaturated fatty acid biosynthesis, as well as in β‐alanine metabolism (Figure 3h). Diterpenoids, derived from (*E,E,E*)‐geranylgeranyl diphosphate (GGPP), serve as precursors for essential compounds, including phytohormone gibberellins (Hu et al. 2021; Ueguchi‐Tanaka 2023). Notably, gibberellins with tetracyclic diterpenoid backbones have been identified as key drivers of MRDD development (Deng et al. 2024; Ueguchi‐Tanaka 2023).

Given the central role of phytohormones in plant immunity (Deng et al. 2024), we next focused on hormone‐related DEGs. In total, 56 DEGs were linked to hormone biosynthesis, metabolic and signalling transduction pathways, including 14 in gibberellins (GA), 18 in auxin (IAA), 4 in cytokinins (CKs), 1 in jasmonic acid (JA), 2 in salicylic acid (SA), 3 in brassinolide (BR), 4 in abscisic acid (ABA) and 14 in ethylene (ETH) (Figures S3a, S4a, S5a,c, S6a). RT‐qPCR validation confirmed concordant expression patterns for 32 phytohormone‐associated DEGs (GA: 11, IAA: 13, CKs: 3, JA: 1, SA: 1, BR: 1, ABA: 3) (Figures S3b, S4b, S5b,d–g and S6b).

### 2.4 Proteomic Profiling of *Zmnpsn13* in Response to RBSDV Infection

To determine whether the altered gene expression in *Zmnpsn13* #1 translates to protein‐level changes, we performed quantitative proteomics on *Zmnpsn13* #1 and WT plants at 24 dpi using a 4D fast data‐independent acquisition approach. This analysis identified 41,672 unique peptides corresponding to 10,681 proteins. Data reliability was supported by violin plots of protein abundance and high Pearson correlation coefficients (Figure S7a,b). Principal component analysis (PCA) further confirmed strong reproducibility among replicates and clear separation between *Zmnpsn13* #1 and WT (Figure S7c).

Applying a threshold of *p*‐value < 0.05 and fold change ≥ 1.5, we detected 1186 differentially expressed proteins (DEPs), including 656 downregulated and 530 upregulated in *Zmnpsn13* #1 relative to WT (Figure S7d,e). As expected, ZmNPSN13 was detected in WT but absent in *Zmnpsn13* #1 plants, while ZmGDIα abundance remained unchanged (Figure S7f–h). Parallel analysis of eight viral proteins further validated the dataset's reliability, as demonstrated by reproducible violin plots, Pearson correlation coefficients and clustering in PCA (Figure S8a–c). Heatmap analysis revealed significantly reduced abundance of seven viral proteins in *Zmnpsn13* #1, with the notable exception of the effector P7‐1, which remained unaffected (Figure S8d–f).

To elucidate the potential roles of DEPs, we performed domain enrichment, subcellular localization, Gene Ontology (GO), Clusters of Orthologous Groups (COG/KOG) and KEGG pathway analyses. Domain enrichment highlighted cytochrome P450, peroxidase, xylanase inhibitor N/C‐terminal and glutathione S‐transferase C‐terminal domains among ZmNPSN13‐regulated DEPs (Figure S9a). Subcellular localization indicated that most DEPs resided in chloroplasts (45.45%), cytoplasm (20.07%) and nuclei (15.09%) (Figure S9b), suggesting that ZmNPSN13 may influence diverse cellular processes.

GO analysis classified DEPs into three categories: (1) Molecular functions (394 DEPs): Dominant activities included oxidoreductase, acid phosphatase and serine‐type exopeptidase/carboxypeptidase activities; (2) Cellular component (477 DEPs): Primary localizations were protein‐containing complexes, cell periphery and intracellular anatomical structures, with notable enrichment in vacuoles, aligning with ZmNPSN13's role in membrane trafficking regulation; (3) Biological process (459 DEPs): Key processes included defence response to insects, cellular response to UV light and negative regulation of growth, with significant enrichment in hormone response pathways (Figure S10a), highlighting *Zmnpsn13*‐mediated alteration of hormone signalling pathways.

COG/KOG analysis further showed DEPs' involvement in post‐translational modification, protein turnover, chaperone activity, signal transduction, intracellular trafficking, secretion, vesicular transport, transcription and carbohydrate transport and metabolism (Figure S10b). KEGG pathway analysis revealed DEPs' enrichment in phenylpropanoid biosynthesis, benzoxazinoid biosynthesis, sesquiterpenoid and triterpenoid biosynthesis and diterpenoid biosynthesis pathways (Figure S10c).

### 2.5 Integrative Transcriptome and Proteome Analysis Reveals Key Pathways Influenced by *ZmNPSN13* During RBSDV Infection

RBSDV infection triggered substantial transcriptomic and proteomic changes in the upper leaves of *Zmnpsn13* #1 plants. To assess the relationship between these alterations, we performed correlation analysis between quantified DEGs and DEPs, identifying 10,138 gene‐protein pairs (Figure S11a). A weak positive correlation (*R* = 0.23) was observed between transcript and protein changes (Figure S11b), suggesting that post‐transcriptional regulation, translational control, post‐translational modifications and protein turnover collectively contribute to the observed discordance between mRNA levels and protein abundance.

**FIGURE 4:**
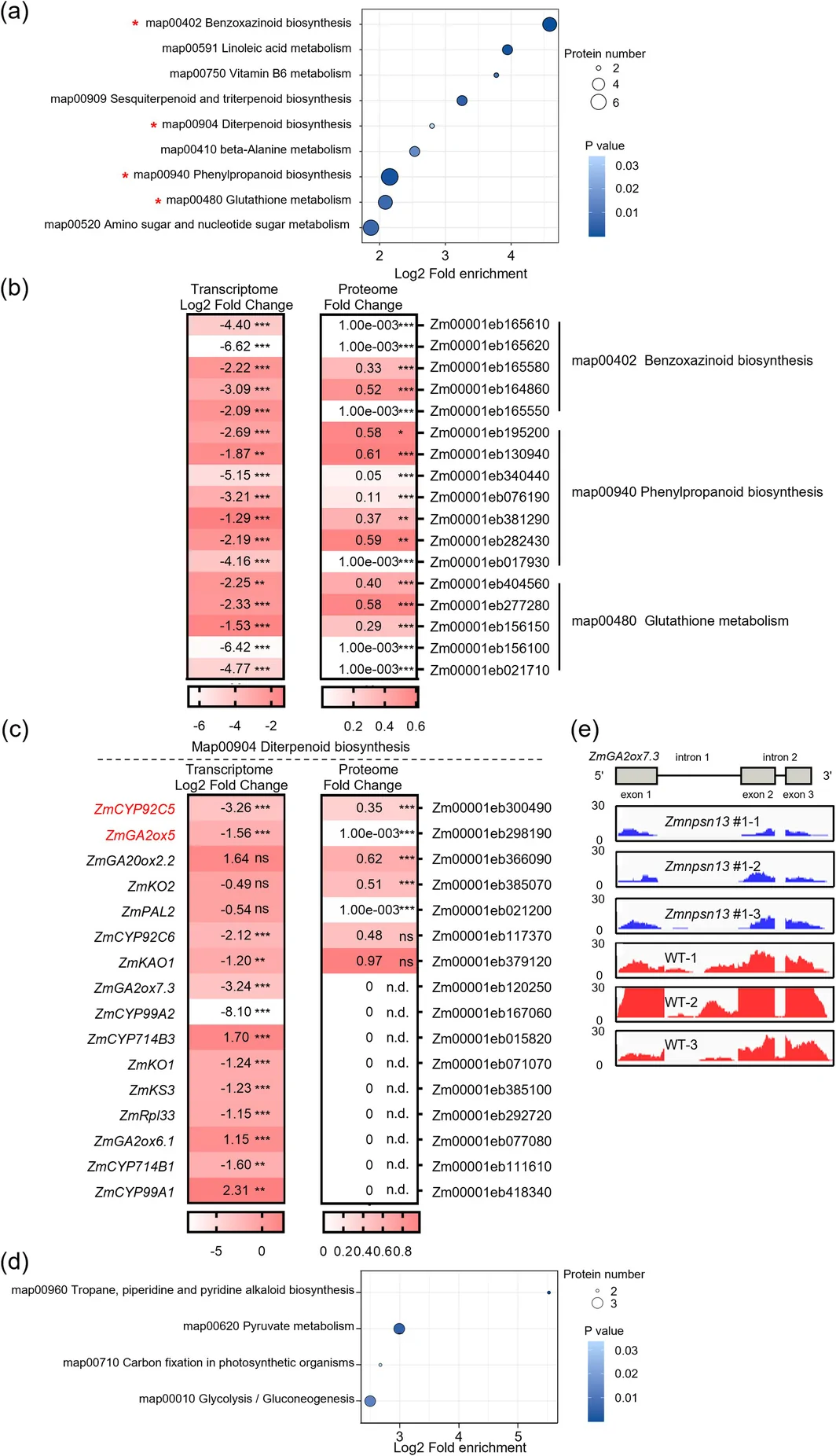
Integrated analysis of rice black‐streaked dwarf virus (RBSDV)‐induced differentially expressed genes (DEGs) and differentially expressed proteins (DEPs) in *Zmnpsn13* #1 versus wild‐type (WT) plants. (a) KEGG analysis of RBSDV‐induced co‐downregulated DEG‐ and DEP‐enriched pathways in *Zmnpsn13* #1 versus WT plants. The circle size represents the number of DEPs. The red asterisks indicate four significantly enriched pathways (*p* < 0.05) involved in biotic stress response. (b) Heatmap of co‐downregulated genes associated with phenylpropanoid biosynthesis, benzoxazinoid biosynthesis and glutathione metabolism at both mRNA and protein levels (*p < 0.05, **p < 0.01, ***p < 0.001). (c) Heatmap of all genes related to diterpenoid biosynthesis at both mRNA and protein levels. The red font indicates two genes significantly downregulated at both levels. (**p < 0.01, ***p < 0.001). (d) KEGG analysis of RBSDV‐induced co‐upregulated DEG‐ and DEP‐enriched pathways in *Zmnpsn13* #1 versus WT plants. The circle size represents the number of DEPs. (e) Enrichment of the *ZmGA2ox7.3* transcript in *Zmnpsn13* #1 versus WT. The gene structure of *ZmGA2ox7.3* is shown at the top. Statistical significance was determined by a two‐tailed Student's t test (b, c).

A subset of 154 genes exhibited coordinated downregulation at both transcriptional and protein levels in *Zmnpsn13* #1 (Figure S11c). GO analysis revealed significant enrichment in glucose‐6‐phosphate transport, hexose phosphate transport, acid phosphatase activity and vacuolar components (Figure S11d–f). KEGG pathway analysis indicated predominant involvement in phenylpropanoid, benzoxazinoid and diterpenoid biosynthesis, along with glutathione metabolism (Figure 4a). Notably, phenylpropanoids, benzoxazinoids and glutathione play critical roles in abiotic stress defence and redox homeostasis maintenance (Abramov et al. 2021; Noctor et al. 2024; Pratyusha and Sarada 2022). All associated genes were significantly downregulated at both mRNA and protein levels in *Zmnpsn13* #1 (Figure 4b).

The diterpenoid biosynthesis pathway, previously implicated in MRDD development (Deng et al. 2024; Ueguchi‐Tanaka 2023), exhibited complex regulation. *ZmCYP92C5* and *ZmGA2ox5* were consistently downregulated at both transcriptional and protein levels, whereas *ZmGA20ox2.2*, *ZmKO2* and *ZmPAL2* were significantly downregulated only at the protein level, likely reflecting post‐translational regulation (Figure 4c). In contrast, while *ZmCYP92C6* and *ZmKAO1* were transcriptionally downregulated, their protein levels remained unchanged. Additionally, nine DEGs were undetectable at the protein level, possibly due to low abundance (Figure 4c).

Conversely, 79 genes exhibited significant upregulation at both the transcriptional and protein levels in *Zmnpsn13* #1 (Figure S11c). GO enrichment analysis revealed their involvement in cellular responses to high‐light intensity, organelle envelope and protein homodimerization activity (Figure S11g–i), while KEGG analysis linked these genes to glycolysis/gluconeogenesis and pyruvate metabolism (Figure 4d).

### 2.6 *ZmNPSN13* Knockout Stabilizes Hormone Homeostasis

Despite substantial reductions in protein abundance, ZmCYP92C5, ZmGA2ox5, ZmGA20ox2.2, ZmKO2 and ZmPAL2 were not detected in our previous co‐precipitation assays with ZmGDIα‐GFP or ZmGDIα‐hel‐GFP (Deng et al. 2024), indicating that they are unlikely to interact directly with ZmNPSN13 (Figure 4c). In contrast, *ZmGA2ox7.3* was significantly downregulated in *Zmnpsn13* #1 at the transcript level, and its protein was undetectable in the proteome (Figure S3a,b and Figure 4e). Given that ZmGA2ox7.3 binds strongly to ZmGDIα and P7‐1 but weakly to ZmGDIα‐hel, and plays a key role in MRDD symptom development by modulating multiple phytohormone pathways (Deng et al. 2024), we hypothesize a potential interaction of ZmGA2ox7.3 with ZmNPSN13.

**FIGURE 5:**
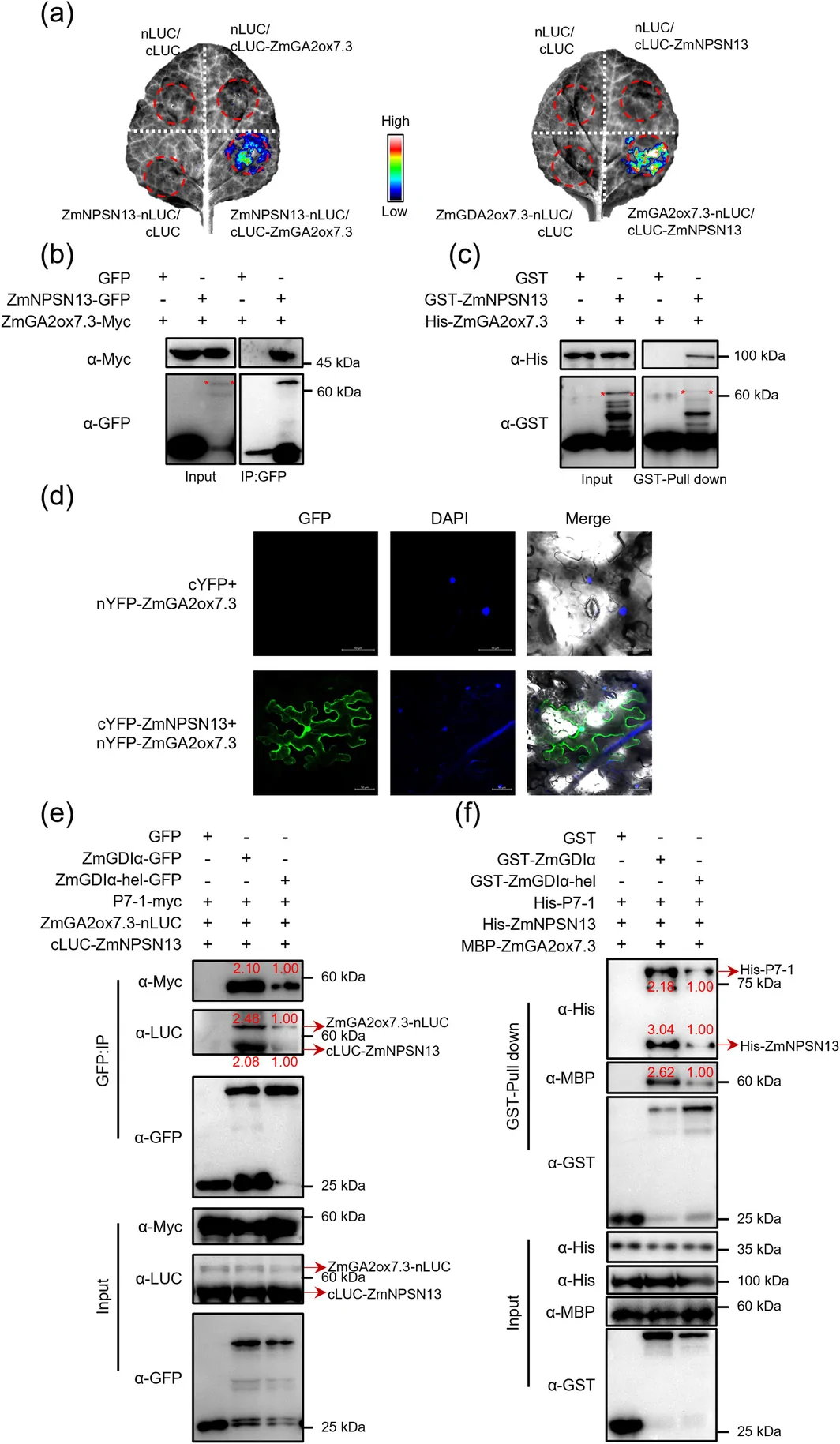
Interactions between ZmNPSN13 and ZmGA2ox7.3. (a) Validation of the interaction between ZmNPSN13 and ZmGA2ox7.3 using an in vivo split luciferase complementation (SLC) assay. The colour scale depicts the intensity of the luminescence signals. (b) Confirmation of the interaction between ZmNPSN13 and ZmGA2ox7.3 using a co‐immunoprecipitation (Co‐IP) assay. (c) Confirmation of the interaction between ZmNPSN13 and ZmGA2ox7.3 using an in vitro glutathione S‐transferase (GST) pull‐down assay. (d) Confirmation of the interaction between ZmNPSN13 and ZmGA2ox7.3 using an in vivo bimolecular fluorescence complementation (BiFC) assay. Reconstituted YFP fluorescence signals were visualized using confocal microscopy at 72 h post‐inoculation (hpi). DAPI staining marks nuclei. Scale bar, 50 μm. (e) Confirmation of the interaction between P7‐1, ZmGDIα/ZmGDIα‐hel, ZmNPSN13 and ZmGA2ox7.3 using a Co‐IP assay. (f) Confirmation of the interaction between P7‐1, ZmGDIα/ZmGDIα‐hel, ZmNPSN13 and ZmGA2ox7.3 using an in vitro GST pull‐down assay.

As expected, MST assays confirmed ZmGA2ox7.3 bound to ZmNPSN13, which was independently validated by SLC, Co‐IP and in vitro pull‐down assays (Figures 1g and 5a–c). Furthermore, BiFC assays localized this interaction to the nucleus, cytoplasm and plasma membrane (Figure 5d). Given the interplay among P7‐1, ZmGDIα/ZmGDIα‐hel, ZmNPSN13 and ZmGA2ox7.3, we hypothesized that these proteins form a pathogenic complex to facilitate MRDD development. To test this, we performed Co‐IP assays in *N. benthamiana* leaves with all four proteins. Both ZmGDIα and ZmGDIα‐hel co‐immunoprecipitated P7‐1, ZmNPSN13 and ZmGA2ox7.3, with stronger interactions observed for ZmGDIα (Figure 5e). Pull‐down assays further confirmed that P7‐1, ZmNPSN13 and ZmGA2ox7.3 bind more strongly to ZmGDIα than to ZmGDIα‐hel (Figure 5f). Collectively, these findings establish ZmNPSN13 as a key component of this pathogenic complex and suggest its role in regulating hormone homeostasis in *ZmNPSN13* knockout mutants.

Previous research indicated that ZmGA2ox7.3 oligomerization significantly enhances its enzymatic activity, facilitating conversion of bioactive GAs into inactive forms (Deng et al. 2024). Consistent with this, RBSDV infection caused extensive transcriptional alterations in hormone biosynthesis and signalling pathways in *ZmNPSN13* #1 (Figures S3a, S4a, S5a,c, S6a). To directly test the impact on phytohormones, we quantified GAs, CKs and auxins in *Zmnpsn13* #1 and WT seedlings infested with viruliferous planthoppers at the emergence stage, with upper leaves collected at 24 dpi.

**FIGURE 6:**
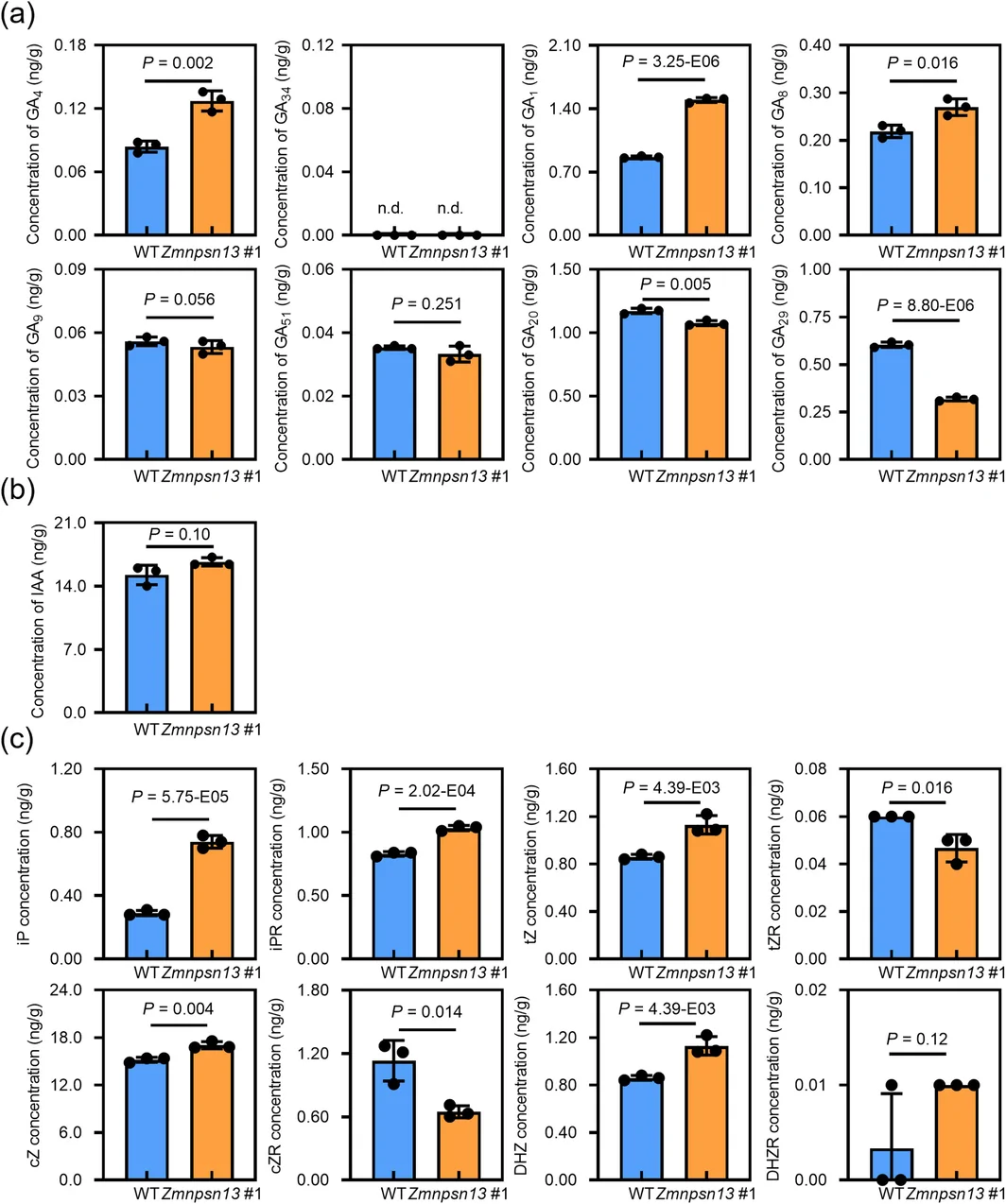
Quantification of endogenous gibberellins, indole acetic acid (IAA) and cytokinins in the upper leaves of *Zmnpsn13* #1 versus wild‐type (WT) plants. (a) Levels of eight gibberellins in the upper leaves of WT and *Zmnpsn13* #1 plants at 24 days post‐inoculation (dpi). (b) Levels of IAA in the upper leaves of WT and *Zmnpsn13* #1 plants at 24 dpi. (c) Levels of eight cytokinins in the upper leaves of WT and *Zmnpsn13* #1 plants at 24 dpi. In (a, b, c), data are presented as mean ± SD with individual data points, as determined by a two‐tailed Student's t test.

Metabolic profiling revealed distinct patterns for eight GA species associated with C_19_‐GA2ox enzymes, including ZmGA2ox7.3. Compared to WT controls, *Zmnpsn13* #*1* plants accumulated significantly higher levels of bioactive GA_1_ and GA_4_, whereas inactive GAs showed variable changes: GA_20_ and GA_29_ were significantly reduced, GA_9_ and GA_51_ remained unchanged, GA_8_ was slightly elevated, and GA_34_ was barely detectable (Figure 6a). These findings demonstrate that *Zmnpsn13* #1 counters gibberellin changes, resembling *ZmGDIα‐hel*, to contribute MRDD resistance.

Given that auxin/CK imbalance underlies white waxy enation formation during RBSDV infection (Deng et al. 2024; Liu et al. 2020), we also assessed auxin and cytokinin levels. While IAA levels were slightly higher in the *Zmnpsn13* #1 mutant than that in WT plants, the difference was not statistically significant (Figure 6b). In contrast, the concentrations of five cytokinins (iP, iPR, tZ, cZ and HZD) were significantly elevated in *Zmnpsn13* #1, whereas tZR and cZR levels decreased significantly, and DHZR remained unchanged (Figure 6c).

Altogether, these results indicate that *ZmNPSN13* knockout alleviates RBSDV‐induced perturbations in growth‐promoting phytohormones, primarily through the upregulation of two bioactive GAs (GA_1_ and GA_4_) and five CKs (iP, iPR, tZ, cZ and HZD). This coordinated hormonal reprogramming, particularly stabilization of GA and CK homeostasis, appears to be a key factor underlying enhanced MRDD resistance observed in *Zmnpsn13* mutants.

### 2.7 A Working Model for *ZmNPSN13* ‐Mediated Negative Regulation of MRDD Resistance

**FIGURE 7:**
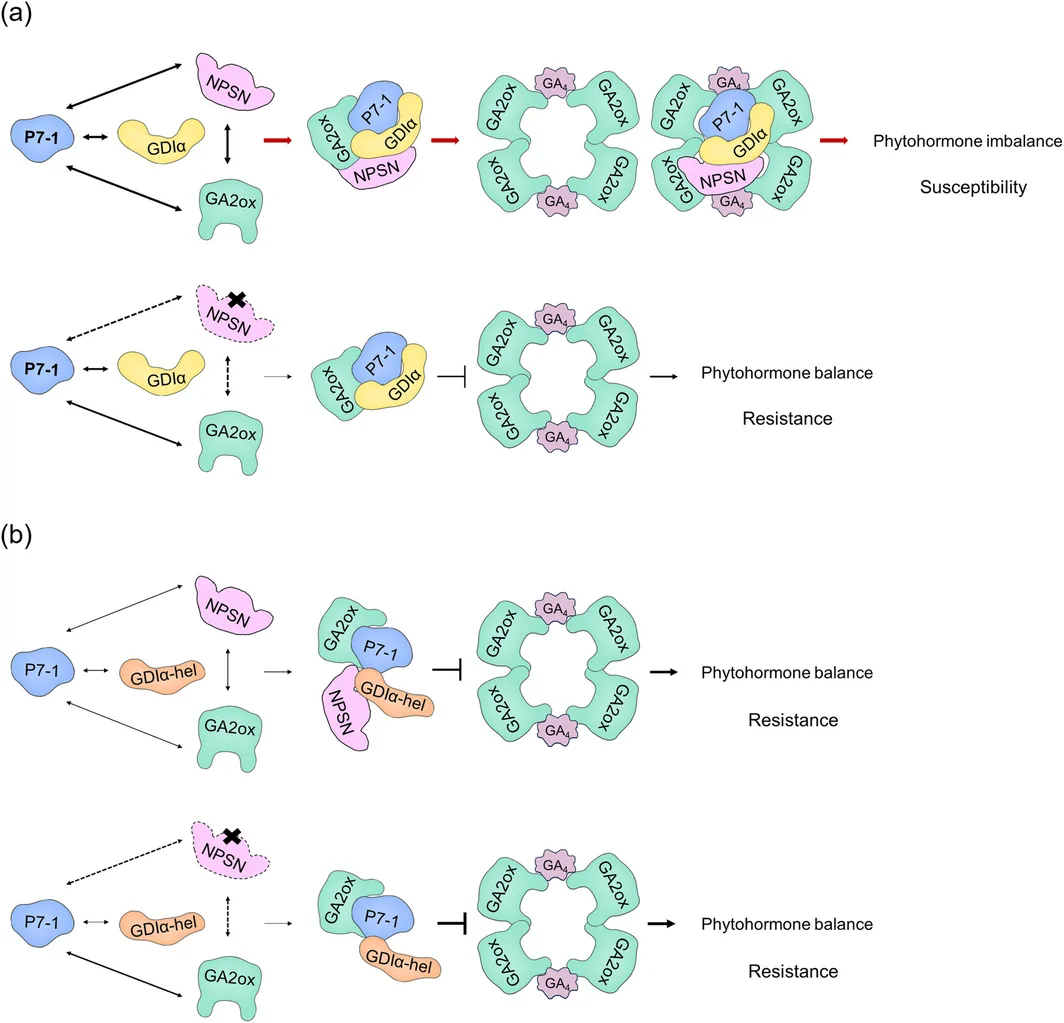
A working model for ZmNPSN13‐mediated negative regulation of maize rough dwarf disease (MRDD) resistance. (a) Top: ZmNPSN13 interacts with the viral P7‐1 effector, the host protein ZmGA2ox7.3 and the target ZmGDIα, forming a pathogenic complex. In rice black‐streaked dwarf virus (RBSDV)‐infected NIL‐S plants (*ZmGDIα*), this stable complex enhances the enzymatic activity of ZmGA2ox7.3, disrupting phytohormone homeostasis and leading to MRDD symptoms. Bottom: In NIL‐S plants, knocking out the *ZmNPSN13* gene suppresses the formation of the pathogenic complex, alleviating RBSDV‐induced hormonal dysregulation and conferring resistance to MRDD. (b) Top: ZmNPSN13 exhibits weakened binding affinity for ZmGDIα‐hel. In RBSDV‐infected NIL‐R plants (*ZmGDIα‐hel*), this weakened interaction decreases the enzymic activity of ZmGA2ox7.3, stabilizing phytohormone homeostasis and conferring resistance to MRDD. Bottom: In NIL‐R plants, knocking out the *ZmNPSN13* gene further suppresses the formation of the pathogenic complex, alleviating RBSDV‐induced hormonal dysregulation and enhancing resistance to MRDD.

We propose a model in which ZmNPSN13 potentiates RBSDV‐induced MRDD development by acting as a key host susceptibility target. ZmNPSN13 facilitates viral infection and compromises MRDD resistance through its interactions with the viral P7‐1 effector, the host susceptibility factor ZmGDIα and the GA‐inactivating enzyme ZmGA2ox7.3, collectively forming a pathogenic complex. In susceptible NIL‐S plants (carrying *ZmGDIα*), RBSDV infection drives the assembly of this complex, elevating the enzymatic activity of ZmGA2ox7.3. This disrupts phytochrome homeostasis, particularly the balance of GAs and CKs, thereby driving MRDD development (Figure 7a). By contrast, in resistant NIL‐R plants (carrying *ZmGDIα‐hel*), ZmNPSN13 exhibits reduced binding affinity for ZmGDIα‐hel, destabilizing complex formation and reducing ZmGA2ox7.3 activity. This preserves phytohormone balance and mitigates MRDD development. Notably, *ZmNPSN13* knockout alleviates RBSDV‐induced hormonal dysregulation, enhancing MRDD resistance, particularly in combination with the *ZmGDIα‐hel* variant (Figure 7b).

## 3 Discussion

Plant SNAREs play key roles in responses to biotic and abiotic stresses (Kwon et al. 2020), yet the functions of *NPSN* (*Qb*‐*SNARE*) genes in plant–pathogen interactions remain poorly understood. In this study, we identify ZmNPSN13, a Qb‐SNARE protein, as a key host susceptibility factor that promotes RBSDV infection and exacerbates MRDD symptoms. ZmNPSN13 interacts with the viral effector P7‐1, the host susceptibility factor ZmGDIα and the GA‐inactivating enzyme ZmGA2ox7.3, forming a pathogenic complex that disrupts phytohormone homeostasis (Figures 1b–g, 5a–f). Notably, replacement of *ZmGDIα* with its resistant variant *ZmGDIα‐hel* destabilizes this complex, mitigates hormonal imbalances and confers MRDD resistance (Figure 1b–f and Figure S1a–g). Moreover, knockout of *ZmNPSN13* alleviated RBSDV‐induced hormonal dysregulation, significantly enhancing MRDD resistance (Figure 6a–c). These findings highlight the pivotal role of SNARE‐mediated vesicle trafficking in plant–virus interactions and provide a molecular basis for breeding MRDD‐resistant maize cultivars.

Mounting evidence indicates that viral effectors often hijack host vesicle trafficking machinery to facilitate infection (Laliberté and Zheng 2014; Zhang et al. 2021). The RBSDV effector P7‐1 emerges as a key virulence determinant, as evidenced by its distinctive early and sustained high expression during infection (Xu et al. 2015). Notably, P7‐1 overexpression in *Arabidopsis* disrupts normal development and induces male sterility, underscoring its pathogenic potential (Sun, Yuan, et al. 2013). Our study reveals that ZmNPSN13 binds more strongly to ZmGDIα and P7‐1 than to ZmGDIα‐hel, suggesting that the stability of the ZmGDIα/P7‐1/ZmNPSN13 complex is crucial for RBSDV pathogenesis (Figure 5e,f). Both overexpression and knockout studies confirmed that *ZmNPSN13* negatively regulates MRDD resistance, further supporting its role as a host target exploited by P7‐1 (Figure 2a–i). At the cellular level, P7‐1 uses host secretory and actomyosin transport systems to target plasmodesmata and orchestrate viral tubule formation, facilitating intracellular viral movement (Sun, Zhang, et al. 2013). Interestingly, *ZmNPSN13* knockout maintains wild‐type levels of both ZmGDIα and P7‐1 proteins during RBSDV infection, suggesting that ZmNPSN13 operates downstream of the initial ZmGDIα/P7‐1 module (Figures S7g,h and S8f). We hypothesize that ZmNPSN13‐mediated vesicle trafficking supports the intracellular transport of RBSDV replication complexes, a role reminiscent of other SNARE proteins in plant–pathogen systems, which can be either exploited by pathogens or contribute to host defence (Collins et al. 2003; Zhu et al. 2019). For instance, *GhSNAP33* overexpression in *Arabidopsis* results in elevated resistance to *Verticillium dahliae* (Wang et al. 2018).

Integrated transcriptomic, proteomic and phytohormone assays revealed that ZmNPSN13 knockout alleviates RBSDV‐induced perturbations in growth‐promoting phytohormones, including two GAs and five CKs (Figures 3h, 6a–c and Figure S10c). This restoration of hormonal balance reinforces ZmGDIα‐hel‐mediated MRDD resistance. Our data suggest that ZmNPSN13 interacts with ZmGA2ox7.3 to promote GA inactivation, thereby disrupting hormonal homeostasis (Figure 6a). The maintenance of GA and CK homeostasis in *Zmnpsn13* #1 likely underlies their enhanced resistance to MRDD (Figure 6a,c). This mechanism parallels findings in *Arabidopsis*, where VAMP727 and SYP22 regulate defence against root‐knot nematodes, potentially by modulating intracellular trafficking of BRI1 (Zhu et al. 2019). These results align with our previous work demonstrating that ZmGA2ox7.3‐mediated GA inactivation drives MRDD symptom development (Deng et al. 2024). Together, the interplay between vesicle trafficking machinery and phytohormone signalling uncovered here establishes a mechanistic link between cellular transport processes and disease susceptibility.

The identification of ZmNPSN13 as a susceptibility factor provides a promising target for molecular breeding. Knockout of *ZmNPSN13* confers strong resistance without apparent pleiotropic effects, highlighting its potential to create optimal resistance alleles for effective MRDD control. Moreover, the recessive nature of *ZmGDIα‐hel*‐mediated resistance, combined with the additive effect observed in *Zmnpsn13* mutants, highlights the potential of stacking multiple resistance alleles for enhanced MDRR resistance (Liu et al. 2020). Future studies could explore whether natural allelic variation in *ZmNPSN13* exists in maize germplasm, which could be harnessed through marker‐assisted selection.

In summary, our study elucidates a mechanism by which RBSDV exploits ZmNPSN13 to subvert vesicle trafficking and disrupt phytohormone homeostasis, leading to MRDD. These findings not only advance our understanding of plant–virus interactions but also provide a foundation for engineering MRDD‐resistant maize varieties through targeted genetic modifications. Future research should focus on investigating the broader roles of SNARE proteins in plant immunity and exploring additional components of the pathogenic complex as potential targets for disease control.

## 4 Experimental Procedures

### 4.1 Transgenic Lines and Plant Materials

For overexpression construct, the full‐length coding sequences of the *ZmNPSN13* was cloned into the pBXCUN vector under control of the maize *Ubiquitin* promoter (UBI), resulting in *Ubi::ZmNPSN13* construct. For the CRISPR/Cas9 knockout construct, the target site located at the fifth exons of *ZmNPSN13* was obtained and cloned into the vector pBUE411 (Xing et al. 2014). All resulting constructs were confirmed by sequencing and transformed into *Agrobacterium tumefaciens* EAH105. The immature zygotic embryos (12 day after pollination) of the maize inbred line B73 were used for *Agrobacterium*‐mediated maize transformation to generate transgenic lines (Ishida et al. 2007; Lee and Zhang 2014). The primer sequences are listed in Table S1.

*ZmNPSN13*‐overexpressing transgenic plants (*ZmNPSN13*‐OE) and *ZmNPSN13* loss‐of‐function mutants (*ZmNPSN13*‐KO) were generated at the Center for Functional Genomics and Molecular Breeding of Crops, China Agricultural University, Beijing. Additionally, the susceptible maize inbred line Huangzao 4 and the resistant inbred line X178, used in our previous study (Liu et al. 2020; Zuo et al. 2015), were employed as recurrent parents.

### 4.2 Artificial Inoculation of RBSDV and Symptom Evaluation

Maize kernels were germinated in vermiculite‐filled pots in a greenhouse. Seedlings at the emergence stage were artificially inoculated with RBSDV‐carrying planthoppers for 3 days, then transplanted into the field, following previously described protocols (Deng et al. 2024; Liu et al. 2020).

As previously study, the typical severity of MRDD can be classified into five grades (0, 0.25, 0.5, 0.75 and 1) base on onset symptoms at the mature stage, particularly dwarfing (Liu et al. 2020), where grade 0 (resistance): normal plants without any symptoms; 0.25: plant height reduced to three‐quarters of that of the normal plant; 0.5: plant height/internodes shortened to two‐third of those of the normal plant; 0.75: severe shortening of plant height/internodes to one‐half of those of the normal plant, accompanying with white waxy enations on the upper leaves and abnormal ear/tassel; grade 1 (high susceptibility): severely stunted plant with extremely shortened internodes, waxy enations on the upper leaves and no ear/tassel (Liu et al. 2020). The disease severity index (DSI) was used to quality MRDD severity, and was calculated as follows:

DSI (%) = (grade × number of plants in grade) × 100/(1 × total number of plants).

### 4.3 Plasmid Construction

To generate *ZmNPSN13‐nLUC*, *cLUC‐ZmNPSN13*, *GST‐ZmNPSN13*, *His‐ZmNPSN13*, *ZmNPSN13‐GFP* and *cYFP‐ZmNPSN13* constructs, the *ZmNPSN13* coding sequence was cloned into JW772‐35S‐NLuc (nLUC), JW771‐35S‐CLuc (cLUC), pGEX6P‐1 (GST), pET28a (His), Super1300‐GFP and P2YC (cYFP) vectors, respectively. In our prior study, the four target genes (*ZmGDIα*, *ZmGDIα‐hel*, *S7‐1* and *ZmGA2ox7.3*) were inserted into JW772‐35S‐Cluc, JW772‐35S‐NLuc, Super1300‐Myc, pGEX6P‐1, pET40 or pCold‐TF vectors, respectively (Deng et al. 2024). To generate the *P7‐1‐mCherry* construct, the full‐length coding sequence of P7‐1 was cloned into the Super1300‐mCherry vector. All primer sequences are listed in Table S1.

### 4.4 SLC Assay

The pair of constructs was co‐infiltrated into *N. benthamiana* leaves, as described previously (Liu et al. 2020). Three days post‐infiltration, leaves were injected with 1 mM luciferin (Promega, E1601) and chemiluminescent signals were captured using the Chemiluminescent Imaging System (Tanon). Total protein was extracted from the infiltrated leaves and analysed by immunoblotting using an α‐LUC antibody (Abcam) and a plant‐specific actin antibody (ABclonal Technology). Fluorescence signals and protein band intensities were quantified using ImageJ Launcher software (National Institutes of Health), with measurements expressed as mean grey values.

### 4.5 Co‐IP Assay

Co‐IP assay was performed as previously described with modifications (Deng et al. 2024). The relevant constructs were transformed into *Agrobacterium tumefaciens* EHA105 and co‐expressed in *N. benthamiana* leaves for 72 h after infiltration. Total proteins were extracted from the infiltrated leaves using IP buffer (50 mM Tris–HCl, 150 mM NaCl, 0.2% Triton X‐100, 5 mM dithiothreitol [DTT], 10 mM PMSF, pH 7.5). Supernatants were used for input detection, and the reminders were incubated with anti‐GFP magnetic beads (MBL) for 2 h at 4°C, followed by three washes with phosphate‐buffered saline (PBS; 0.137 M NaCl, 2.68 mM KCl, 4 mM Na_2_HPO_4_, 1.76 mM KH_2_PO_4_, pH 7.4). The immunoprecipitated complexes were heated in 1× SDS loading buffer (Genstar) at 100°C for 5 min and analysed by immunoblotting using anti‐LUC antibody (Abcam), anti‐GFP and anti‐Myc antibodies (ABclonal Technology).

### 4.6 Pull‐Down Assay

The relevant fusion constructs were transformed into *Escherichia coli* BL21 (DE3) (TransGen Biotech). Recombinant proteins (GST‐ZmNPSN13, His‐ZmNPSN13, GST‐ZmGDIα, GST‐ZmGDIα‐hel, His‐ZmGDIα, His‐ZmGDIα‐hel, His‐ZmGA2ox7.3, MBP‐ZmGA2ox7.3 and His‐P7‐1) were purified by affinity chromatography, in accordance with the manufacturers' instructions. GST or GST‐tagged proteins were immobilized on glutathione agarose beads at 4°C for 1 h, followed by incubation with His‐tagged or MBP‐tagged proteins for an additional 2 h at 4°C. Bound protein complexes were eluted and detected by immunoblotting using anti‐GST, anti‐His and anti‐MBP antibodies (Easybio).

### 4.7 BiFC Assay

Paired constructs were transformed into *Agrobacterium tumefaciens* EHA105 and co‐infiltrated into *N. benthamiana* leaves for 72 h. GFP fluorescence was visualized using confocal microscopy (ZEISS880, Carl Zeiss) with an excitation wavelength of 488 nm.

### 4.8 Subcellular Localization and BFA Treatment

The *ZmNPSN13‐GFP*, *P7‐1‐mCherry*, *GFP* and *35S:AtMAN1‐mCherry* (a marker for ER, Golgi apparatus and plasma membrane) constructs were transformed into *N. benthamiana* leaves by *Agrobacterium‐*mediated infiltration. The initial infiltration sites were injected with 50 μM BFA for 6 h. The GFP and mCherry fluorescence signals were visualized using confocal laser scanning microscopy (ZEISS880, Carl Zeiss) after 72 h of transformation. The fluorescence intensities were quantified using ZEN software.

### 4.9 MST Assay

The purified proteins, GST‐ZmNPSN13, His‐P7‐1, His‐ZmGA2ox7.3, His‐ZmGDIα and His‐ZmGDIα‐hel, were concentrated and dialysed in PBS buffer using an Amicon Ultra‐4 concentrator unit with a 10 kDa molecular weight cut‐off (Millipore). In brief, 25 nM GST‐tagged ZmNPSN13 protein was labelled with RED‐NHS dye (Nano Temper Technologies, MO‐L011) and then incubated with serial dilutions of ZmGA2ox7.3, P7‐1, ZmGDIα or ZmGDIα‐hel ligands. The binding reactions were measured by using a microscale thermophoresis instrument (Nano Temper Technologies) at 25°C, 40% MST power and 20% LED power and the dissociation constant (K_d_) was calculated by MO affinity Analysis software.

### 4.10 RNA Extraction, RT‐qPCR and RNA‐Seq

Total RNA was extracted from the upper leaves of maize lines B73, OE‐1 to OE‐7, WT and *Zmnpsn13* #1 to 4 using the PLANTpure Plant RNA Kit (Aidlab). The RNA was then reverse‐transcribed into cDNA using TransScript RT/RI reverse transcriptase with random primers (TransGen Biotech). Gene expression levels were qualified using RT‐qPCR performed on a CFX Connect real‐time RT‐PCR system (Bio‐Rad) using a TB Green qPCR kit (Takara). The maize tubulin gene *ZmTubulin* served as an internal control. Primer sequences used for qPCR are listed in Table S1. Each experiment was conducted with at least three biological replicates.

RNA‐seq was performed by Novogene Bioinformatics Technology (Beijing, China). Three biological replicates were independently prepared for the mRNA‐seq libraries, using the entire uppermost leaves harvested from at least five individual plants per sample. RNA quality was evaluated using a NanoDrop spectrophotometer and an Agilent 2100 Bioanalyzer. Sequencing libraries were prepared and sequenced on an Illumina Hiseq platform (125/150 bp paired‐end). Raw reads were processed to remove adapters and low‐quality bases, and then aligned to the maize B73 RefGen_v5 genome using HISAT2 (v2.0.5).

Gene expression levels were quantified as FPKM (fragments per kilobase of transcript per million mapped reads) using HTSeq (v. 0.6.1). Differential expression analysis was performed using DESeq (v. 1.20.0) with thresholds of log_2_FC > |1| and *p* < 0.05. Gene Ontology (GO) enrichment and KEGG pathway enrichment analyses were conducted using clusterProfiler (v. 3.8.1). Data visualization was performed with IGV (v. 2.16.1).

### 4.11 Proteomics Analysis

Three biological replicates of leaves (each from 10 individual plants) of WT and *Zmnpsn13* #1 plants were sampled. Protein extraction, digestion and LC–MS/MS analysis were performed by PTM BIO (Hangzhou, China). Proteins were extracted in lysis buffer (1% Triton X‐100, 100 mM DTT and 1% protease inhibitor cocktail, 50 μM PR‐619, 3 μM trichostatin A, 50 mM nicotinamide, 2 mM EDTA and 1% phosphatase inhibitor for phosphorylation). Proteins were precipitated by slowly adding trichloroacetic acid to a final concentration of 20% (w/v).

The precipitated proteins were then subjected to tryptic digestion. The resulting peptides were desalted using a Strata X SPE column and separated on an EASY‐nLC 1200 UPLC system (ThermoFisher Scientific). The separated peptides were analysed in an Orbitrap Exploris 480 with a nano‐electrospray ion source. MS/MS spectra were analysed with Proteome Discoverer 1.3 (Thermo Electron) coupled with the MASCOT engine (Matrix Science) against the Uniprot database.

### 4.12 Measurements of Plant Hormone Content

WT and *Zmnpsn13* #1 plants were artificially infested with RBSDV‐carrying planthoppers. Upper leaves were sampled at 24 days post‐infestation and used to measure the levels of various endogenous phytohormones, including GAs, IAA and CKs. Phytohormone quantification was performed using UHPLC–MS/MS analysis (Thermo Scientific Ultimate 3000 UHPLC coupled with TSQ Quantiva) as described by Chen et al. (2012). This analysis was conducted by Wuhan Greensword Creation Technology Co. Ltd. (http://www.greenswordcreation.com). Each experiment included three biological replicates.

### 4.13 Quantification and Statistical Analysis

Statistical analysis was performed using GraphPad Prism 8. One‐way ANOVA Tukey's test and a two‐tailed Student's t test were used to calculate *p* values. Significant differences (*p* < 0.05) are indicated by different letters or asterisks above the bars. Details of the experimental design and statistical analysis are provided in the respective figure legends.

## Author Contributions

**Suining Deng:** writing – original draft, software, methodology, formal analysis, data curation, resources, writing – review and editing, conceptualization, investigation, validation. **Yixiao Qin:** methodology. **Baoshen Liu:** methodology. **Siqi Jiang:** methodology. **Tao Zhong:** methodology, writing – original draft. **Qingcai Liu:** methodology. **Jianju Liu:** methodology. **Yuanliang Liu:** methodology. **Manman Li:** methodology. **Yanmei Li:** methodology. **Mingzhu Yan:** methodology. **Qian Li:** writing – original draft. **Mingliang Xu:** writing – review and editing, writing – original draft, funding acquisition, visualization, project administration, resources, supervision.

## Funding

This work was supported by Biological Breeding‐Major Projects, 2023ZD04070. Breeding Research and Development Program, NK20230702.

## Conflicts of Interest

The authors declare no conflicts of interest.

## Supporting information


**Figure S1:** ZmNPSN13 binds more tightly to ZmGDIα and P7‐1 than to ZmGDIα‐hel.

**Figure S2:** Subcellular localization of ZmNPSN13 and P7‐1.

**Figure S3:** Differentially expressed genes involved in gibberellin‐related pathways.

**Figure S4:** Differentially expressed genes involved in auxin signalling pathways.

**Figure S5:** Differentially expressed genes involved in other hormone‐related pathways.

**Figure S6:** Differentially expressed genes involved in ethylene‐related pathways.

**Figure S7:** Proteomic analysis of differentially expressed proteins (DEPs) between *Zmnpsn13* #1 and wild‐type (WT) plants in response to RBSDV infection.

**Figure S8:** Proteomic analysis of viral protein expression in *Zmnpsn13* #1 versus wild‐type (WT) plants upon RBSDV infection.

**Figure S9:** Domain enrichment and subcellular localization analysis of differentially expressed proteins.

**Figure S10:** Comparative proteomics profiling of RBSDV‐induced differentially expressed proteins (DEPs) in *Zmnpsn13* #1 versus wild‐type (WT) plants.

**Figure S11:** Integrated transcriptomic and proteomic analysis of Zmnpsn13 #1 and wild‐type (WT) plants in response to RBSDV infection.

**Table S1:** A list of primers used in the research.

**Table S2:** Samples size for transgenic validation in the research.

## Data Availability

The data that support the findings of this study are available on request from the corresponding author. The data are not publicly available due to privacy or ethical restrictions.
