# Supplementary material for: ZmNPSN13 Potentiates Maize Rough Dwarf Disease Upon Recruitment by the RBSDV P7‐1 Effector
Source: Mol Plant Pathol. 2026 May 19;27(5):e70277. doi: 10.1111/mpp.70277 (PMC13581069; doi:10.1111/mpp.70277)
Supplement: Supplementary file 1 [file MPP-27-e70277-s002.docx]

**Figure S10.** Comparative proteomics profiling of RBSDV-induced differentially expressed proteins (DEPs) in *Zmnpsn13* #1 versus WT plants.

(a) GO classification of DEPs-enriched terms in biological processes (BP), cellular components (CC), and molecular functions (MF), with circle sizes representing the number of DEPs. The red lines indicate significantly enriched processes and asterisks denote response to hormone pathway.

(b) COG/KOG categorization of DEPs enrichment, highlighting cellular processes/signaling (red), information storage/processing (green), metabolism (blue), and poorly characterized functions (yellow). The red asterisks indicate the five enriched COG/KOG category.

(c) KEGG analysis of RBSDV-induced DEPs in *Zmnpsn13* #1 revealed enriched pathways, where circle sizes represent the number of DEPs. Three significantly enriched pathways (*P* < 0.05), marked by red asterisks, showed consistency with transcriptome data.
