# Supplementary material for: ZmNPSN13 Potentiates Maize Rough Dwarf Disease Upon Recruitment by the RBSDV P7‐1 Effector
Source: Mol Plant Pathol. 2026 May 19;27(5):e70277. doi: 10.1111/mpp.70277 (PMC13581069; doi:10.1111/mpp.70277)
Supplement: Supplementary file 2 [file MPP-27-e70277-s004.docx]

**Figure S1.** ZmNPSN13 binds more tightly to ZmGDIα and P7-1 than to ZmGDIα-hel.

(a) Interactions of ZmNPSN13 with ZmGDIα, ZmGDIα-hel, or P7-1 in SLC assays. The protein combinations were as follows: ZmNPSN13/ZmGDIα (I-1, I-4), ZmNPSN13/ZmGDIα-hel (I-2), and ZmNPSN13/P7-1 (I-3). Luciferase activity was measured at 72 hours post-inoculation (hpi).

(b, c) Quantification of protein-protein interaction strength based on relative luminescence intensity. Signal intensities were measured using ImageJ software (b), with statistical analysis of luminescence intensities (c). Data are depicted as individual points (n = 8) within boxes.

(d–f) Quantification of exogenously expressed proteins. Exogenous proteins ZmGDIα-nLUC, ZmGDIα-hel-nLUC, and P7-1-nLUC were detected through immunoblotting using an anti-LUC antibody (d), while cLUC-ZmNPSN13 across four combinations was detected using the same antibody (e). Total protein loading was assessed using an anti-actin antibody (f). Each point represents a biological replicate (n = 8).

(g) Normalized quantification of protein-protein interaction strength based on relative luminescence intensity and protein abundance (ZmGDIα-nLUC, ZmGDIα-hel-nLUC, and P7-1-nLUC). Data are depicted as individual points (n = 8) within boxes.

In (c, d, e and g), error bars represent the range from the minimum to maximum values, with medians marked by central lines. Box edges denote the 25^th^ and 75^th^ percentiles. Statistical significance was determined by one-way ANOVA with Tukey’s test (c, d, e and g).
