# Supplementary material for: ZmNPSN13 Potentiates Maize Rough Dwarf Disease Upon Recruitment by the RBSDV P7‐1 Effector
Source: Mol Plant Pathol. 2026 May 19;27(5):e70277. doi: 10.1111/mpp.70277 (PMC13581069; doi:10.1111/mpp.70277)
Supplement: Supplementary file 3 [file MPP-27-e70277-s005.docx]

**Figure S5.** DEGs involved in other hormone-related pathways.

(a, c) Heatmaps displaying DEGs involved in CKs (a), JA, SA, BR and ABA (c) in *Zmnpsn13* #1 versus WT. DEGs are displayed by a color gradient scale, with red indicating upregulation and blue indicating downregulation (fold change > 2.0, *P* < 0.05).

(b and d–g) Relative expression levels of DEGs associated with CKs, JA, SA, BR, and ABA verified by qRT-PCR. Genes exhibiting consistent expression patterns between RNA-seq and qRT-PCR are marked in red. WT was served as the control.

In (b and d–g), data are presented as means ± s.e.m (four biological replicates). Statistical significance was determined using a two-tailed Student’s *t*-test.
