# Supplementary material for: ZmNPSN13 Potentiates Maize Rough Dwarf Disease Upon Recruitment by the RBSDV P7‐1 Effector
Source: Mol Plant Pathol. 2026 May 19;27(5):e70277. doi: 10.1111/mpp.70277 (PMC13581069; doi:10.1111/mpp.70277)
Supplement: Supplementary file 4 [file MPP-27-e70277-s006.docx]

**Figure S4.** DEGs involved in auxin signaling pathways.

(a) Heatmap displaying DEGs associated with auxin signaling in *Zmnpsn13* #1 versus WT. DEGs are displayed using a color gradient scale, with red indicating upregulation and blue indicating downregulation (fold change > 2.0, *P* < 0.05).

(b) Relative expression levels of 18 auxin-related DEGs verified by qRT-PCR. Genes exhibiting consistent expression patterns between RNA-seq and qRT-PCR are marked in red. WT was served as the control.

In (b), data are presented as means ± s.e.m. (four biological replicates). Statistical significance was determined using a two-tailed Student’s *t*-test.
