# Supplementary material for: ZmNPSN13 Potentiates Maize Rough Dwarf Disease Upon Recruitment by the RBSDV P7‐1 Effector
Source: Mol Plant Pathol. 2026 May 19;27(5):e70277. doi: 10.1111/mpp.70277 (PMC13581069; doi:10.1111/mpp.70277)
Supplement: Supplementary file 5 [file MPP-27-e70277-s007.docx]

**Figure S9.** Domain enrichment and subcellular localization analysis of DEPs.

(a) Enriched protein domains among DEPs. The circle size represents the number of DEPs. The red asterisks indicate five significantly enriched domains.

(b) Subcellular localization of DEPs. Outside ring: Classification and proportional distribution of subcellular compartments. Inner circle: Differential expression ratio (*Zmnpsn13* vs. WT) for each compartment, with upregulated (red) and downregulated (blue) proteins expressed as percentages
