# Supplementary material for: ZmNPSN13 Potentiates Maize Rough Dwarf Disease Upon Recruitment by the RBSDV P7‐1 Effector
Source: Mol Plant Pathol. 2026 May 19;27(5):e70277. doi: 10.1111/mpp.70277 (PMC13581069; doi:10.1111/mpp.70277)
Supplement: Supplementary file 6 [file MPP-27-e70277-s008.docx]

**Figure S8.** Proteomic analysis of viral protein expression in *Zmnpsn13* #1 versus WT plants upon RBSDV infection.

(a) Violin plots comparing viral protein expression distributions in *Zmnpsn13* versus WT plants. Solid horizontal lines indicate medians, box limits represent interquartile ranges (IQR), and whiskers extend to 1.5× IQR.

(b) Pearson correlation matrix of viral protein expression across biological replicates.

(c) Three-dimensional Principal Component Analysis (PCA) of viral protein profiles from *Zmnpsn13* #1 and WT plants post-RBSDV infection.

(d) Heatmap of viral DEPs in *Zmnpsn13* versus WT.

(e) Normalized abundance of RBSDV P7-1 protein in *Zmnpsn13* #1 versus WT.

(f) Western blot validation of RBSDV P7-1 protein levels.

In (e and f), data are presented as means ± s.d. (n = 3). Statistical significance was determined using a two-tailed Student’s *t*-test.
