# Supplementary material for: ZmNPSN13 Potentiates Maize Rough Dwarf Disease Upon Recruitment by the RBSDV P7‐1 Effector
Source: Mol Plant Pathol. 2026 May 19;27(5):e70277. doi: 10.1111/mpp.70277 (PMC13581069; doi:10.1111/mpp.70277)
Supplement: Supplementary file 8 [file MPP-27-e70277-s010.docx]

**Figure S7.** Proteomic analysis of differentially expressed proteins (DEPs) between *Zmnpsn13* #1 and WT plants in response to RBSDV infection.

(a) Violin plots comparing protein expression distributions in *Zmnpsn13* versus WT plants.

(b) Pearson correlation matrix of biological repeats. Solid horizontal lines indicate medians, box limits represent interquartile ranges (IQR), and whiskers extend to 1.5× IQR.

(c) Three-dimensional Principal Component Analysis (PCA) of WT and *Zmnpsn13* after RBSDV infection (n= 3 replicates per group). PC = Principal Component.

(d) Volcano plot of DEPs in *Zmnpsn13* versus WT at 24 dpi. Proteins with significant downregulation are represented by blue dots, those with significant upregulation by red dots, and those without significant changes by gray dots.

(e) Heatmap of DEPs in *Zmnpsn13* versus WT.

(f) Normalized abundance of *ZmNPSN13* protein in *Zmnpsn13* #1 versus WT.

(g) Normalized abundance of ZmGDIα protein in *Zmnpsn13* #1 versus WT.

(h) Western blot validation of ZmGDIα protein levels.

In (f–h), data are presented as means ± s.d. (n =3). Statistical significance was determined by a two-tailed Student’s *t*-test.
