# Supplementary material for: ZmNPSN13 Potentiates Maize Rough Dwarf Disease Upon Recruitment by the RBSDV P7‐1 Effector
Source: Mol Plant Pathol. 2026 May 19;27(5):e70277. doi: 10.1111/mpp.70277 (PMC13581069; doi:10.1111/mpp.70277)
Supplement: Supplementary file 10 [file MPP-27-e70277-s012.docx]

**Figure S2. Subcellular localization of ZmNPSN13 and P7-1.**

(a) *N. benthamiana* leaves were co-transformed AtMAN1-mCherry with the control vector (above) and the ZmNPSN13-GFP (bottom). respectively. AtMAN1 serves as a marker for endoplasmic reticulum, Golgi apparatus and plasma membrane. The white frame represents the colocalization of ZmNPSN13-GFP and AtMAN1 in Golgi apparatus. Scale bars, 50 μm. The white asterisks represent the Golgi apparatus. The fluorescence density profile was measured along the while line using ZEN software.

(b) Effect of BFA on the subcellular localization of ZmNPSN13-GFP in *N. benthamiana* leaves. Scale bars, 50 μm. The white asterisks represent the BFA bodies.

(c) P7-1-mCherry colocalizes with ZmNPSN13-GFP in the BFA bodies in *N. benthamiana* leaves. The white asterisks represent the BFA bodies. The fluorescence density profile was measured along the while line using ZEN software. Scale bars, 50 μm.
