# Supplementary material for: ZmNPSN13 Potentiates Maize Rough Dwarf Disease Upon Recruitment by the RBSDV P7‐1 Effector
Source: Mol Plant Pathol. 2026 May 19;27(5):e70277. doi: 10.1111/mpp.70277 (PMC13581069; doi:10.1111/mpp.70277)
Supplement: Supplementary file 11 [file MPP-27-e70277-s013.docx]

**Figure S11.** Integrated transcriptomic and proteomic analysis of *Zmnpsn13* #1 and WT plants in response to RBSDV infection.

(a) Venn diagram showing overlap between DEGs and DAPs.

(b) Correlation heatmap of changes in mRNA and protein abundances (*Zmnpsn13* vs. WT).

(c) Venn diagram of the overlapped upregulated (or downregulated) DEGs and DEPs.

(d–f) GO enrichment analysis of co-downregulated DEGs/DEPs enriched in biological processes (d), molecular functions (e), and cellular components (f).

(g–i) GO enrichment analysis of co-upregulated DEGs/DEPs enriched in biological processes (g), molecular functions (h), and cellular components (i).
